# Bone marrow disease in rhabdomyosarcoma visualized by 2-[^18^F]fluorodeoxyglucose positron emission tomography/computed tomography

**DOI:** 10.1007/s00247-024-05933-5

**Published:** 2024-04-26

**Authors:** Pia Kröning, Sebastian Berg, Martin T Freitag, Reineke A Schoot, Alexandra Fischer, Alexander Puzik, T Feuchtinger, Charlotte Niemeyer, Philipp Tobias Meyer, Markus Uhl, Simone Hettmer

**Affiliations:** 1 Division of General Pediatrics, Department of Pediatric and Adolescent Medicine, University Medical Center Freiburg, University of Freiburg, Freiburg, Germany; 2 Division of Pediatric Radiology, Department of Radiology, University Medical Center Freiburg, University of Freiburg, Freiburg, Germany; 3 Department of Nuclear Medicine, University Medical Center Freiburg, University of Freiburg, Freiburg, Germany; 4 Princess Máxima Center for Pediatric Oncology, Utrecht, the Netherlands; 5 Division of Pediatric Hematology and Oncology, Department of Pediatric and Adolescent Medicine, University Medical Center Freiburg, University of Freiburg, Freiburg, Germany; 6 Universitätsmedizin Halle, Martin Luther University, Pediatrics 1, Ernst-Grube-Strasse 40, 06120, Halle (Saale), Germany

**Keywords:** Bone marrow metastases, Flurodeoxyglucose F18, Pediatrics, Positron emission tomography computed tomography, Rhabdomyosarcoma

## Abstract

Bone marrow metastases—noted in 6% of patients with rhabdomyosarcoma—have been linked to very poor outcomes. Bilateral bone marrow sampling from iliac crests has been the gold standard for bone marrow examination in rhabdomyosarcoma, but sampling errors due to patchy bone marrow involvement may limit its sensitivity. Here, we report the case of a 6-year-old boy with embryonal rhabdomyosarcoma of the skull base and multiple 2-[^18^F]fluoro-2-deoxy-D-glucose (2-[^18^F]FDG)-avid bone marrow metastases visualized by positron emission tomography and computed tomography (2-[^18^F]FDG PET/CT). His bone marrow aspirates were tumor-free. This case illustrates the diagnostic value of 2-[^18^F]FDG PET/CT in the detection of bone marrow metastases in rhabdomyosarcoma patients, which may re-shape the definition of bone marrow disease and, ultimately, alter disease staging and risk stratification.

## Introduction

Rhabdomyosarcoma is the most common soft tissue sarcoma of adolescence and childhood. The two main pediatric subtypes are embryonal rhabdomyosarcoma and alveolar rhabdomyosarcoma. Morphology, molecular characteristics, and clinical behavior are diverse [1]. Approximately 6% of all patients with rhabdomyosarcoma have bone marrow metastases at the time of diagnosis, which predicts an aggressive course of disease. Estimated 3-year event-free survival is 14% compared to 34% in patients with metastatic disease but no bone marrow involvement [2]. The intensity of multimodal rhabdomyosarcoma treatment is stratified based on a number of risk factors, including disease stage [1]. Bilateral bone marrow sampling from iliac crests has been the gold standard for bone marrow examination as an important component of rhabdomyosarcoma staging and risk assessment. Recently, the International Soft Tissue sarcoma Consortium (INSTRuCT) recommended that 2-[^18^F]fluoro-2-deoxy-D-glucose positron emission tomography co-registered to computed tomography (2-[^18^F]FDG PET/CT) also be performed during primary rhabdomyosarcoma staging [3]. 2-[^18^F]FDG PET/CT is reported to detect primary tumors, lymph node, and bone metastases in pediatric patients with sarcomas with > 90% sensitivity, and appears to be especially sensitive in detecting increased 2-[^18^F]FDG uptake at skeletal disease sites [4–7]. The striking potential of 2-[^18^F]FDG PET/CT imaging in detecting bone marrow metastases is illustrated by the case described here in which bone marrow aspirates at the time of rhabdomyosarcoma diagnosis were tumor-free, but multiple 2-[^18^F]FDG-avid bone marrow metastases were detected by 2-[^18^F]FDG PET/CT. Lack of cortical destruction and absence of a soft tissue mass (as visualized by the CT component of the scan) indicated isolated bone marrow involvement. Informed consent was obtained from the patient and his parents for publication of this case report.

## Case report

**Fig. 1:**
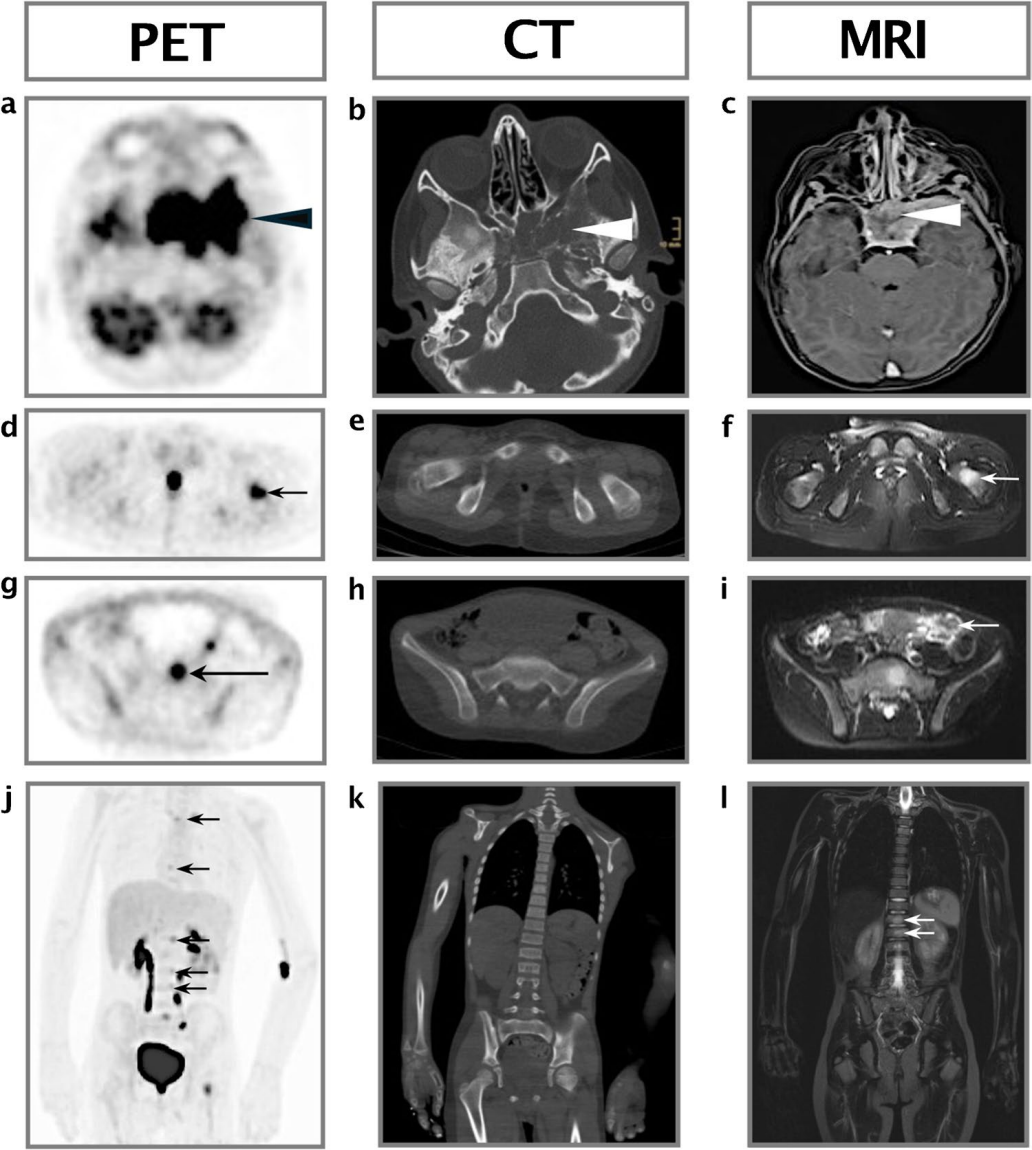
Findings in 2-[^18^F]fluoro-2-deoxy-D-glucose positron emission tomography (2-[^18^F]FDG-PET), computed tomography (CT) and magnetic resonance imaging (MRI) at the time of diagnosis in a 6-year-old boy with metastatic rhabdomyosarcoma. **a**–**c** Axial plane 2-[^18^F]FDG PET image (**a**) shows the large primary tumor at the skull base with 2-[^18^F]FDG uptake above the mediastinal blood pool (*arrowhead*). Axial plane CT image viewed on bone windows (**b**) and axial plane T2-weighted contrast MRI image (**c**) show the primary tumor, which extends from the sella turcica into the fossa infratemporalis and is associated with a large soft tissue mass (*arrowheads*). **d**–**f** Axial plane 2-[^18^F]FDG PET image (**d**) shows a 2-[^18^F]FDG-avid lesion in the left femur (*arrow*). Axial plane CT image viewed on bone windows (**e**) and axial plane T2-weighted MRI image without contrast (**f**) do not reveal changes in trabecular bone structure or an associated soft tissue mass (*arrow*). **g**–**i** Axial plane 2-[^18^F]FDG PET image (**g**) demonstrates a 2-[^18^F]FDG-avid lesion in the sacrum (*arrow*). Axial plane CT image viewed on bone windows (**h**) and axial plane T2-weighted MRI image without contrast (**i**) do not reveal changes in trabecular bone structure or an associated soft tissue mass (*arrow*). **j**–**l** Coronal plane 2-[^18^F]FDG PET image (**j**) shows 2-[^18^F]FDG-avid foci at thoracic (T) and lumbar (L) vertebrae (T4, T8, L1, L2, and L3). Coronal plane CT image viewed on bone windows (**k**) and T2-weighted MRI without contrast (**l**) do not show changes in trabecular bone structure or associated soft tissue masses (*arrows*)

A 6.5-year-old previously healthy boy presented with ptosis, double vision, and dysesthesias of the left side of the face. Magnetic resonance imaging (MRI, Magnetom Avanto Fit, Siemens, Erlangen, Germany) and computed tomography (CT, Somatom Definition Flash, Siemens, Erlangen, Germany) revealed a large parameningeal mass adjacent to the internal carotid artery, extending from the sella turcica into the fossa temporalis and infratemporalis (Fig. 1). The mass was biopsied, and rhabdomyosarcoma of the embryonal subtype without evidence for a *FOXO1* breakpoint was diagnosed. Staging examinations included MRI of the chest, abdomen, and extremities, chest CT, 2-[^18^F]FDG PET/CT (Gemini TF TOF 64, Philips, Hamburg, Germany), and bilateral bone marrow aspirates obtained from the iliac crests. Microscopic evaluation of bilateral bone marrow aspirates did not reveal infiltration by tumor cells. Bone marrow biopsies were not performed. Multiple 2-[^18^F]FDG-avid foci were detected involving thoracic (T) and lumbar (L) vertebrae (T4, T8, T12, L1, L2, L3) (Fig. 1), the sacrum (Fig. 1), and the left femur (Fig. 1). At these sites, the bone marrow space exhibited foci of increased signal in the fat-saturated T2 MRI sequences (Fig. 1). CT did not reveal any cortical destruction or osteolytic/ osteoblastic alteration of the trabecular bone structure (Fig. 1). No associated soft tissue masses and no additional sites of bone marrow metastases were visualized by MRI (Fig. 1). The lesions were considered bone marrow metastases. Chemotherapy was administered according to standard-of-care recommendations for treatment of metastatic rhabdomyosarcomas and well-tolerated. Restaging after three cycles of chemotherapy revealed an excellent response: the previously detected hypermetabolic metastatic sites in the vertebrae, sacral bone, and left femur were metabolically inactive, and the primary tumor was smaller in size and FDG-negative (images not shown). The patient went on to complete proton beam irradiation of the primary tumor site, nine cycles of intensive chemotherapy, and six cycles of oral maintenance therapy. He continues to be in first complete remission 7.5 years after diagnosis.

## Discussion

Direct bone marrow assessment by microscopic evaluation of bone marrow aspirates and trephine biopsies has been the gold standard in bone marrow staging of children and adolescents diagnosed with cancer. Recently, the need to perform bone marrow biopsies in patients with low-risk rhabdomyosarcoma without local invasion or evidence for lymph node/lung metastases has been called into question, as retrospective analyses demonstrated 0% bone marrow metastases in such patients [7]. Another retrospective assessment revealed detection of bone marrow metastases with 92% sensitivity/94% specificity by 2-[^18^F]FDG-PET/CTs compared to direct bone marrow sampling [8]. Yet, as four of 114 patients had PET/biopsy + bone marrow disease, it was concluded that bone marrow biopsies could not be omitted from the initial staging of patients diagnosed with rhabdomyosarcoma [8]. Alternatively, whole body MRI may allow for non-invasive detection of bone marrow metastases without radiation exposure, but with long imaging times.

The case reported here illustrates detection of bone marrow metastases in a 6-year-old boy with parameningeal rhabdomyosarcoma by combined assessment of 2-[^18^F]FDG-PET/CT and MRI. As none of the 2-[^18^F]FDG-avid bone marrow sites was biopsied directly, the lack of cytological validation of bone marrow disease represents a limitation of this report. Albeit extremely unlikely, we cannot completely rule out other causes of increased 2-[^18^F]FDG uptake such as chronic non-bacterial osteomyelitis or Langerhans cell histiocytosis. When using 2-[^18^F]FDG PET/CT for bone marrow staging, its limitations should be kept in mind: 2-[^18^F]FDG PET/CT imaging is associated with exposure to ionizing radiation, requires anesthesia in young children, does not depict 2-[^18^F]FDG-negative or very small lesions, and may visualize reactive changes in bone marrow activity (e.g., following chemotherapy), which must not be mistaken for tumor spread. Also, distinguishing bone from bone marrow metastases represents a challenge and requires consideration of signs of cortical/trabecular destruction, presence of soft tissue masses, and abnormal bone marrow signal depicted by MRI.

Arguably, these limitations are outweighed by major advantages associated with bone marrow staging by 2-[^18^F]FDG PET/CT: above all, the stress, pain, and need for some form of anesthesia—typically associated with direct bone marrow sampling by bone marrow aspirates/ trephine biopsies—can be avoided. Also, bone marrow metastases may be detected more reliably. Bone marrow infiltration by solid tumor or lymphoma cells is often patchy, and bone marrow staging based on bone marrow aspirates/trephine biopsies (typically obtained from the right and left posterior iliac crests) comes with a degree of sampling error. 2-[^18^F]FDG PET/CT allows depiction of the entire trunk and limbs and may identify sites of bone marrow disease that are otherwise not detectable. This may change disease stage and, consequently, intensity of treatment in some patients. Widespread use of 2-[^18^F]FDG PET/CT in rhabdomyosarcoma staging may ultimately require redefinition of bone marrow disease and its impact on risk stratification.

## Data Availability

Not applicable.
